# First Episode Psychosis: A Neurodevelopmental Crossroads

**DOI:** 10.1192/j.eurpsy.2025.1848

**Published:** 2025-08-26

**Authors:** N. Monteiro, M. Vasconcelos, R. Resende, S. Morais, D. Mota

**Affiliations:** 1 Centro de Responsabilidade Integrada de Psiquiatria, Centro Hospitalar e Universitário de Coimbra (CHUC); 2 Faculdade de Medicina, Universidade de Coimbra, Coimbra, Portugal

## Abstract

**Introduction:**

Autism spectrum disorder (ASD) and schizophrenia (SZ) are neurodevelopmental disorders that, although unfolding in different ways, can present with overlapping symptoms, both negative symptoms like deficits in social–emotional reciprocity and engagement (Trevisan *et al.* Front.Psych; 2020;11:548), and positive symptoms like delusions and hallucinations (Ribolsi *et al.* Front Psychiatry; 2022;13:768586).

**Objectives:**

To discuss the diagnostic challenges between ASD and SZ in patients presenting with both positive and negative symptoms.

**Methods:**

In addition to describing a case report of a man with negative symptoms and presumptive psychotic symptoms, research was undertaken in PubMed and other databases using the keywords “autism spectrum disorder”, “schizophrenia” and “multiple sclerosis”.

**Results:**

A 26 year-old man was involuntarily admitted to the in-patient unit due to persecutory delusions, irritability, social isolation and cognitive symptoms. He had also been recently diagnosed with Multiple Sclerosis (MS). These symptoms had begun 5 years prior, intensifying over time, leading to the hypothesis of First Episode Psychosis, with a probable recent escalation secondary to the flairing up of MS. Through a detailed clinical history, we discovered that, in fact, the patient exhibited conduct changes since early adolescence: restricted and repetitive behavior, social isolation, reduced tolerance to opposition, cognitive rigidity, circumscribed interests and puerile contact. This lead to the development of great hostility towards his family members, whenever his wants weren’t met (most of them mismatched with reality), resulting in isolation from the family and the sending of aggressive messages and emails, even though his parents always tried to provide the patient with everything he wanted, explaining the assumption of persecutory delusions. Intramuscular risperidone and clozapine were initiated for irritability and cognitive symptoms, respectively, with minimal improvement in both, mainting however every other symptom described.

**Conclusions:**

Despite the current distinction between ASD and SZ, they still share many similarities, increasing the difficulty of determining an exact diagnosis. We present a case with negative and cognitive symptoms, that can fit in both conditions, and positive symptoms that fit in SZ. It’s possible to understand that the delusions may not be primary, but secondary to social interpretation bias, common in ASD patients, and that part of the cognitive symptoms can be due to MS. The suboptimal response to antipsychotics also makes us lean more to the presence of ASD with temporary psychotic symptoms instead of a primary psychotic disorder.

**Disclosure of Interest:**

None Declared

